# Epigenetic and Transcriptomic Pathways Underlying Animal Models of Cognitive and Psychiatric Disorders: A Scoping Review

**DOI:** 10.3390/cimb48040425

**Published:** 2026-04-21

**Authors:** Jaishriram Rathored, Ajay Pal, Deepika Sai Painkra

**Affiliations:** 1 Department of Central Research Laboratory and Molecular Diagnostics, Datta Meghe Institute of Higher Education & Research, Sawangi Meghe, Wardha 442001, Maharashtra, India; deepsai266@gmail.com; 2 Centre for Neural Development and Repair (CNDR), Lewis Katz School of Medicine, Temple University, 3500 N. Broad St., Philadelphia, PA 19140, USA

**Keywords:** epigenetics, transcriptomics, DNA methylation, histone modification, autism spectrum disorder, schizophrenia, depression, Rett syndrome, scoping review, inhibitory interneurons, excitatory–inhibitory balance, PV+ interneurons

## Abstract

**Background:** Cognitive and psychiatric disorders are caused by a complex interplay between genetic predisposition, environmental exposures, and dynamic molecular regulation in the brain. Animal models provide a controlled environment for examining these mechanisms, and advances in transcriptome and epigenomic technologies have greatly expanded our knowledge of disease-relevant pathways. **Objective:** This scoping review systematically maps and synthesizes the epigenetic and transcriptomic findings from the established animal models of four neuropsychiatric conditions—autism spectrum disorder (ASD), schizophrenia, depression, and Rett syndrome—drawing on a PRISMA-ScR-guided literature search. The review characterizes the breadth of evidence, identifies convergent and divergent molecular pathways, and highlights the translational gaps and therapeutic implications. **Methods:** Research employing chromatin accessibility testing, genome-wide DNA methylation mapping, single-cell and bulk RNA sequencing, histone modification profiling, and multi-omics integration in mouse and other validated animal models was thoroughly reviewed. A quality appraisal of the primary experimental studies (n = 63) was performed using a modified CAMARADES checklist. **Results:** Beyond generalized cellular stress responses, multi-omics analysis emphasizes the cell-type- and context-dependent nature of epigenetic changes in animal models, including isoform-specific histone modifications and model-dependent binding of HDAC/MeCP2 complexes to genes involved in synaptic plasticity. Single-cell RNA sequencing analyses have uniformly shown transcriptional changes in parvalbumin-positive (PV+) interneurons. **Conclusions:** The specific convergence of epigenetic disruptions in neural circuits involved in synaptic structure and inhibitory function could play a role in the generation of neuropsychiatric phenotypes in animal models, highlighting the importance of circuit- and cell-type-specific epigenetics while pointing to potential therapeutic avenues.

## 1. Introduction

Neuropsychiatric disorders—a broad category encompassing neurodevelopmental conditions such as autism spectrum disorder (ASD) and Rett syndrome, as well as psychiatric disorders including schizophrenia and depression—have a significant impact on public health because of the intricate interactions between genetics, the environment, and molecular mechanisms that influence the genesis of these conditions [1,2,3]. In this review, ‘neuropsychiatric disorders’ is used as the umbrella term; ‘neurodevelopmental disorders’ refers to ASD and Rett syndrome; and ‘psychiatric disorders’ refers to schizophrenia and depression.

While some modes of inheritance can be explained by genetic variation, there is accumulating evidence that genetic background interacts with neural circuitry and epigenetic and transcriptomic regulatory mechanisms to shape neuronal function, which in turn gives rise to behavior [4,5,6]. Epigenetic mechanisms dynamically regulate gene expression and enable neuronal plasticity to respond to developmental factors, environmental changes, and synaptic activity. Examples include non-coding RNA control, histone modifications, and DNA methylation [7,8,9]. Epigenetic regulation ultimately shapes the transcriptional output of neural circuits, linking chromatin-level changes to functional alterations in neuronal networks, through transcriptomic processes including alternative splicing, RNA editing, and regulatory RNA activity [10,11,12,13].

Animal models have been invaluable in deciphering the molecular basis of these diseases, providing researchers with an experimental tool to study pathogenic pathways via genetic manipulation, prenatal exposure paradigms, or chronic stress protocols in rodents and primates [14,15,16,17]. The current state-of-the-art knowledge on epigenetics and transcriptomics in animal models of ASD, schizophrenia, depression, and Rett syndrome is compiled in this scoping review, with emphasis on common molecular mechanisms, novel experimental approaches, divergent findings across model systems, and possible therapeutic applications.

## 2. Materials and Methods

This scoping review was carried out in accordance with the PRISMA extension for Scoping Reviews (PRISMA-ScR; Willsey et al., 2018 [18]) to identify and synthesize key epigenetic and transcriptomic pathways involved in animal models of ASD, schizophrenia, depression, and Rett syndrome.

### 2.1. PRISMA Registration and Compliance

**Figure 1:**
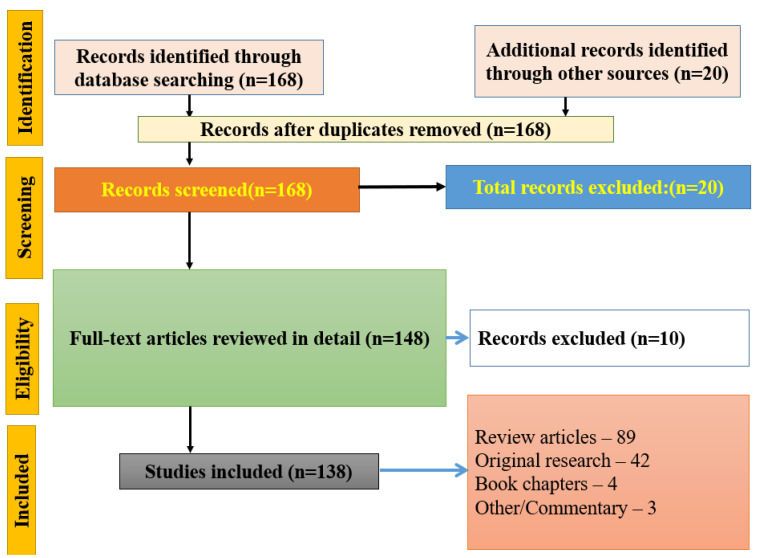
PRISMA-ScR flow diagram (updated to reflect final included corpus of 138 publications).

The Preferred Reporting Items for Systematic Reviews and Meta-Analyses extension for Scoping Reviews (PRISMA-ScR) standards were followed in the conduct and reporting of this scoping review. Scoping reviews are designed to map the breadth of evidence across heterogeneous study designs and evidence types, explicitly permitting the inclusion of primary studies, reviews, and other literature types to characterize the landscape of a field—the methodology that best matches the objectives of the present review. The study protocol was prospectively registered with registration number CRD420251268584 in PROSPERO. A PRISMA flow diagram is shown in Figure 1.

### 2.2. Search Strategy and Study Identification

A total of 168 records were identified through searches of PubMed, Scopus, Web of Science, and Google Scholar, using combinations of: “epigenetics”, “transcriptomics”, “DNA methylation”, “histone modification”, “non-coding RNA”, “animal models”, “autism spectrum disorder”, “schizophrenia”, “depression”, and “Rett syndrome”. An additional 20 records were identified through cross-referencing and manual screening.

### 2.3. Screening and Eligibility

After duplicate removal, 168 records were screened based on their title and abstract. Twenty records were excluded as they were out of scope (non-neuropsychiatric topics, non-English language, or full text unavailable).

### 2.4. Full-Text Assessment

Of the 148 records assessed for full-text eligibility, 10 were excluded because their findings were entirely duplicated by other included publications (the same experimental model, same gene loci, overlapping author teams reporting the same dataset). These excluded articles are listed with explicit reasons in Supplementary Table S2. The remaining 138 publications were included in the scoping review corpus.

### 2.5. Inclusion Criteria

Studies were included if they reported epigenetic and/or transcriptomic mechanisms associated with ASD, schizophrenia, depression, or Rett syndrome, using animal studies, human postmortem/clinical samples, or multimodal approaches. Eligible studies were experimental, mechanistic, or analytical, with respect to DNA methylation, histone modifications, miRNA regulation, or transcriptomic profiling. Review articles, book chapters, and commentaries were also included to contextualize the mechanistic findings, consistent with the scoping review methodology. All of the included literature types are distinguished in Table 1A,B.

### 2.6. Exclusion Criteria

Studies were excluded if they: (1) addressed targets or populations unrelated to the four neuropsychiatric conditions under review; (2) used non-vertebrate systems without established translational relevance; (3) consisted of conference abstracts, case reports, or editorials lacking primary data; or (4) reported findings that were entirely replicated by another included study from the same research group, using the same dataset.

### 2.7. Quality Assessment of Primary Experimental Studies

Primary experimental animal studies (n = 89 original research articles) were appraised using a modified CAMARADES (Collaborative Approach to Meta-Analysis and Review of Animal Data from Experimental Studies) checklist. Items evaluated: (1) Use of randomization in group allocation; (2) blinded outcome assessment; (3) sample size justification; (4) conflict of interest statement; (5) animal housing and welfare conditions; and (6) appropriate statistical methods with effect size reporting. The results are summarized in Supplementary Table S1. This appraisal contextualizes the evidence strength in the narrative; consistent with the scoping review design, it was not used to exclude studies from the corpus.

### 2.8. Evidence Stratification

Evidence is explicitly stratified into three tiers throughout this review: (1) Primary experimental animal studies—the core evidence base; (2) human postmortem or clinical studies—cited for translational context; and (3) secondary literature (reviews, meta-analyses, book chapters)—used to frame concepts and identify gaps. These tiers are labeled in Table 1A,B and indicated in the narrative.

## 3. Results

A total of 188 records were identified: 168 were retrieved through database searching and 20 were from manual cross-referencing. After duplicate removal, 158 records were screened; 20 were excluded at the title/abstract stage. Of the remaining 148 full-text records, 10 were excluded (duplicated datasets; Supplementary Table S2). The final included corpus comprised 138 publications: 89 original research articles, 42 review articles, 4 book chapters, and 3 commentaries [Figure 1]. This composition is consistent with the scoping review methodology.

To facilitate synthesis, the included literature was organized into two evidence tiers. Table 1A presents 28 primary experimental studies selected from the 89 original research articles to represent each of the four disorders and each major methodological approach covered in this review. Selection criteria for Table 1A: (a) Defined animal model with specified species and strain; (b) at least one epigenomic or transcriptomic measurement; and (c) mechanistic rather than purely correlational findings. Table 1B presents 12 key review and conceptual papers that provided theoretical frameworks that were foundational to this field. All 89 original experimental studies are synthesized in the narrative.

### 3.1. Epigenetic Mechanisms in the Brain

**Figure 2:**
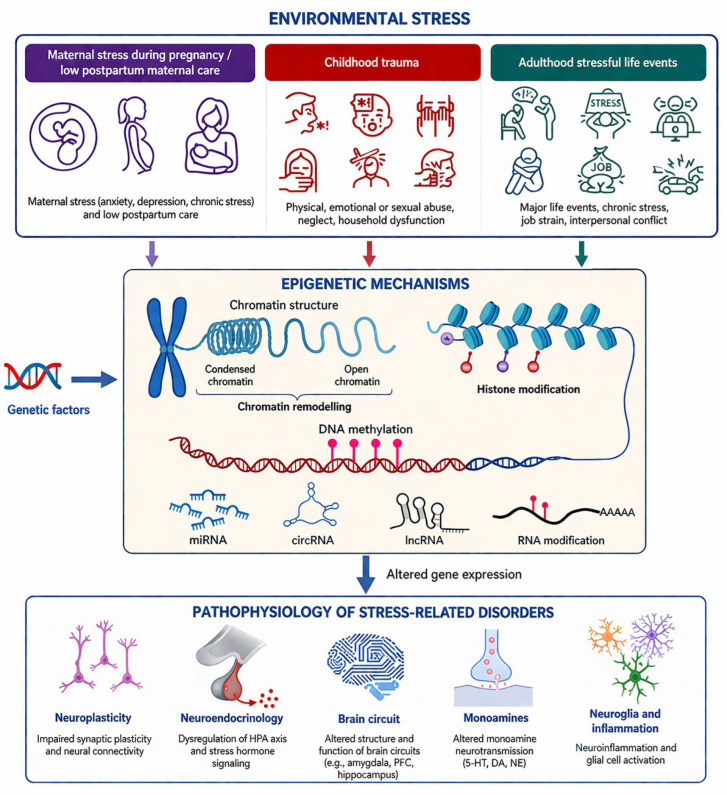
Stress-induced epigenetic and transcriptomic mechanisms underlying the pathophysiology of stress-related brain disorders.

DNA methylation and other epigenetic mechanisms regulate gene expression in neurons and glia. DNA methyltransferases (DNMTs) interact with other chromatin-modifying mechanisms to affect chromatin accessibility and transcriptional activity [31,34,35]. Long-term methylation patterns affecting genes involved in neuronal development, synaptic plasticity, and inhibitory neurotransmission can be significantly altered by early-life developmental and environmental disruptions such as stress, maternal immune activation, and exposure to toxins [23,32,36,37] [Figure 2]. Hypermethylation of both GAD67 and RELN promoters in animal models suggests a connection to the decrease in inhibitory neurons and alterations in cortical excitability, which human brain tissue investigations are verifying [21,38,39]. Multi-omics analyses indicate that transcription factors like AP-1 serve as key nodes integrating transcriptional and epigenetic regulation [40,41,42,43].

Histone acetylation is typically associated with transcription activation, whereas methylation can either activate or repress expression depending on the specific residue and context [17,29,44,45,46]. Decreased histone acetylation at the BDNF promoter in chronic stress models is associated with reduced transcription and depressive-like behaviors; HDAC inhibitor treatment restores chromatin accessibility and improves behavioral outcomes [47,48,49]. Certain histone methylation changes, particularly H3K4me3, are involved in the regulation of synaptic gene expression; their altered regulation has been connected to cognitive deficits in animal models of schizophrenia and ASD through alterations in the excitatory–inhibitory balance in the cortical and hippocampal circuits [17,22,50].

**Figure 3:**
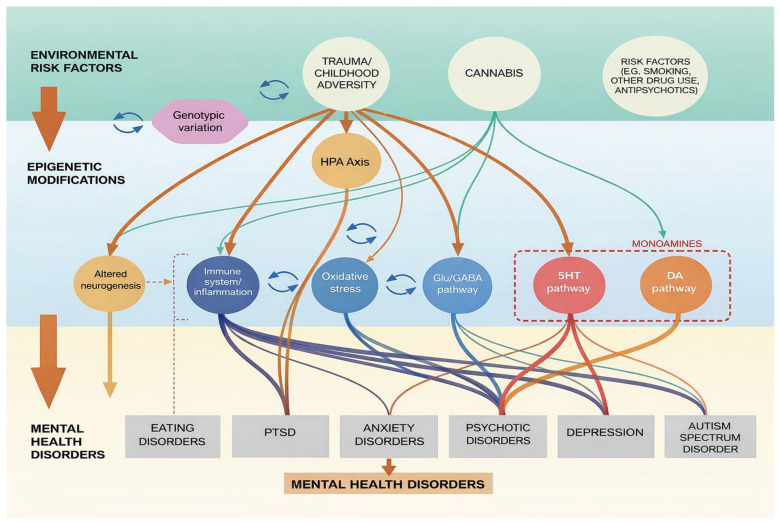
Environmental and epigenetically influenced biological pathways underlying mental health disorders.

MicroRNAs (miRNAs), long non-coding RNAs (lncRNAs), and circular RNAs (circRNAs) support transcriptional and epigenetic regulation by altering the chromatin structure and gene expression. miR-132 and miR-134 affect the synaptic plasticity, neurotrophic factor signaling, and dendritic spine morphology [51,52,53]. Altered miRNA expression profiles in animal models of autism and schizophrenia further upset the excitatory–inhibitory balance and affect synaptic protein expression. lncRNAs such as Neat1 and Malat1 impact social behavior and stress response in mice [54,55,56]. Collectively, these findings highlight how multiple epigenetic regulatory systems interact to affect neural circuit function, enhancing the picture of environmentally influenced biological pathways shown in Figure 3.

### 3.2. Transcriptomic Regulation in Neural Function

**Figure 4:**
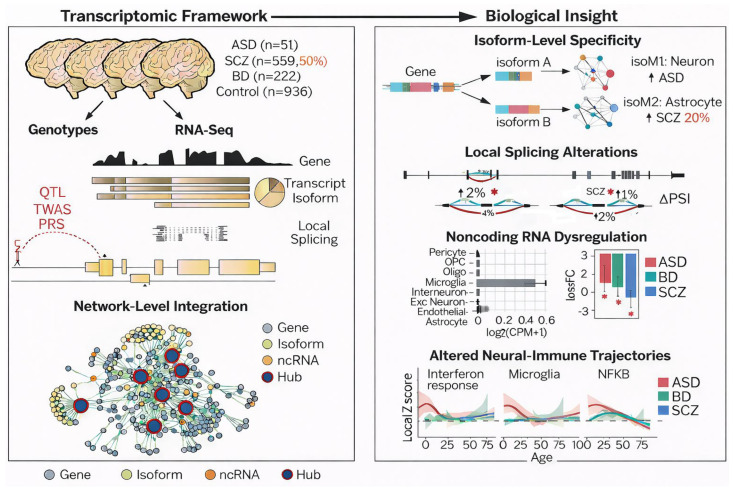
Transcriptomic and splicing-related alterations across major psychiatric disorders, summarizing RNA sequencing-based analyses across ASD, SCZ, and bipolar disorder (BD) and illustrating isoform-level specificity, local splicing alterations, non-coding RNA dysregulation, and network-level integration. This figure illustrates a transcriptomic framework integrating genotypic and RNA-seq data to generate biological insights in autism spectrum disorder (ASD), schizophrenia (SCZ), bipolar disorder (BD), and controls. The left panel shows transcriptomic analyses at the gene, transcript isoform, local splicing, and network-integration levels. The right panel summarizes key biological findings, including isoform-level specificity, local splicing alterations, noncoding RNA dysregulation, and altered neural-immune trajectories. * *p* < 0.05.

Epigenetic drivers provide the regulatory control over molecular communication processes. Alternative mRNA splicing variants generate distinct protein isoforms that are essential for synaptic selectivity. Genes including SHANK3, NRXN1, and DISC1, whose improper splicing causes the excitation–inhibition imbalance in schizophrenia and ASD, have been identified in model organisms [26,57,58] [Figure 4]. Aberrant splicing alters the composition of synaptic scaffolding complexes, neurotransmitter receptor trafficking, and axon–dendrite connectivity, resulting in circuit-level dysfunction within the cortical and hippocampal networks [59,60,61].

RNA editing, especially adenosine-to-inosine (A-to-I) substitutions, affects protein activity by changing specific coding regions of receptors or channels. Disrupted RNA-editing of AMPA and serotonin receptor subunits occurs in schizophrenia models, leading to changes in neurotransmission and synaptic plasticity [62,63,64]. lncRNAs, circRNAs, and miRNAs collaborate to control gene networks involved in neurogenesis, synaptic transmission, and stress response [55,56,57]. Three common pathways of dysregulation—synapse, mitochondria, and inflammation—converge across animal model transcriptomes [65,66,67,68], revealing shared molecular vulnerabilities across various neuropsychiatric conditions.

### 3.3. Animal Models of Autism Spectrum Disorder

**Figure 5:**
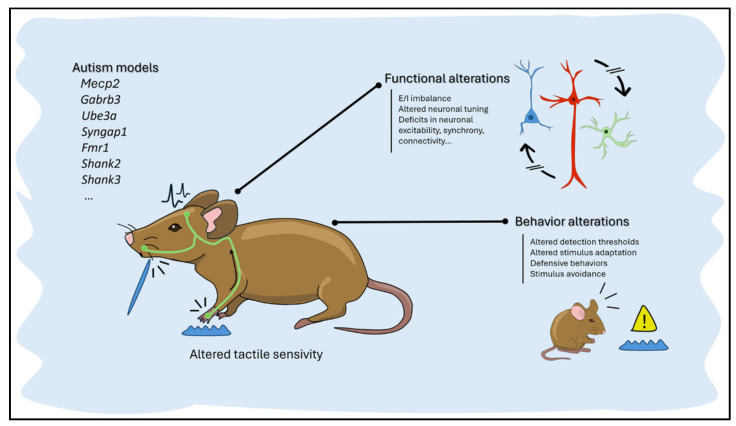
Sensory and circuit disruptions caused by ASD-associated genetic mutations in animal models.

The pathophysiology of ASD has been studied extensively, using animal models [Figure 5]. Shank3 knockout mice, Gabrb3, Mecp2, Ube3a, Syngap1, Fmr1, and Shank2 mutants exhibit impaired sociability, repetitive behaviors, and disturbed synaptic processes [19,69,70]. Decreased histone acetylation and aberrant production of synaptic scaffolding proteins are linked to E/I imbalance and reduced dendritic spine density [71,72,73]. Additional evidence for circuit-level anomalies comes from structural and functional neuroimaging investigations [74,75]. ** Prenatal VPA exposure causes global histone hyperacetylation, oxidative stress, and immunological gene expression changes, leading to autism-like symptoms [76,77]. Epigenetic dysregulation of RELN and GAD67 genes modifies chromatin organization in inhibitory interneurons [20,78,79]. Transcriptomic profiling identifies pathogenic mechanisms, including neuroinflammation, mitochondrial metabolism, synaptic vesicle trafficking, and epigenetic–transcriptomic interactions [80,81,82].

Notably, findings across ASD animal models are not uniform, and a critical comparison reveals important molecular divergences. Shank3 knockout studies report H3K4me3 enrichment at synaptic gene loci—consistent with an upregulated transcription of certain plasticity-related genes—while VPA-exposed models show global histone [83,84] as being model-dependent, rather than convergent at the chromatin level. Similarly, Fmr1 knockout mice show increased mGluR5-dependent synaptic protein synthesis, while Shank2 models exhibit reduced NMDA receptor function [26], pointing to divergent synaptic mechanisms even within the shared ASD phenotypic umbrella. This heterogeneity has direct therapeutic implications: interventions targeting histone acetylation or mGluR5 may be effective in specific model-defined ASD subtypes, but not universally across the spectrum.

From a model validity perspective, genetic ASD models such as Shank3 and Shank2 knockouts have strong construct validity (human SHANK mutations cause Phelan–McDermid syndrome and ASD) and moderate-to-good face validity (social interaction deficits, repetitive behaviors). However, predictive validity remains partial: mGluR5 antagonists normalized Shank3 and Fmr1 mouse phenotypes but failed in clinical trials, highlighting the difficulty of translating circuit-level pharmacology from rodent to human contexts. The VPA model has moderate face validity but weaker construct validity, as prenatal VPA represents only a small fraction of ASD etiologies. Studies focused exclusively on male rodents may not capture the female ASD phenotype, a significant limitation given the known sex differential in ASD presentation.

### 3.4. Animal Models of Schizophrenia

The primary symptoms of schizophrenia are social dysfunction, cognitive impairment, and poor working memory. Many features of the disorder, such as the downregulation of interneuron transcripts and genetic changes at GAD67 and RELN promoters, are replicated in maternal immune activation (MIA) models, using polyinosinic:polycytidylic acid (Poly I:C) for in utero exposure [69,85,86]. Conversely, methylazoxymethanol acetate (MAM) administration disrupts cortical lamination and decreases synaptic gene expression, leading to hypofrontality [83,87,88]. Genetic models—including DISC1 mutation mice and 22q11.2 deletion mice—show altered transcriptional activity of the synaptic, mitochondrial, and inflammatory pathways [27,84,89]. Epigenetic therapy including HDAC inhibitors and LSD1 modulators can ameliorate behavioral abnormalities and partially restore gene expression, identifying shared molecular targets [90,91,92].

A critical comparison across schizophrenia models reveals meaningful molecular divergences. MIA (Poly I:C) and MAM models both reduce GAD67 expression, but via different upstream mechanisms: Poly I:C acts primarily through cytokine-driven DNMT upregulation and subsequent promoter hypermethylation, whereas MAM disrupts GAD67 through altered neurodevelopmental transcription factor programs, rather than direct methylation changes [27,84,89,90]. This distinction is therapeutically important: DNMT inhibitors may be effective in immune-triggered schizophrenia-like states, but may not address the structural origins of GAD67 loss in MAM models. Furthermore, 22q11.2 deletion models show prominent mitochondrial dysfunction and altered calcium homeostasis [93], whereas DISC1 models emphasize synaptic scaffolding and dopaminergic dysregulation, suggesting distinct pathological entry points that converge on shared inhibitory interneuron deficits.

Regarding model validity, the Poly I:C MIA model has moderate construct validity (maternal immune activation is implicated in a proportion of human SCZ cases) and moderate face validity (prepulse inhibition deficits, cognitive impairment). Predictive validity is limited. The MAM model has stronger construct validity for the neurodevelopmental hypothesis of SCZ and replicates electrophysiological hallmarks. The 22q11.2 deletion model is genetically precise (high construct validity). All major SCZ models are disproportionately male, limiting inference to one sex.

### 3.5. Animal Models of Depression

Environmental stressors, including learned helplessness, maternal separation, and chronic social defeat stress (CSDS), impact epigenetics and transcriptomics. Decreased histone acetylation at the BDNF locus and increased promoter methylation of *Crf* and *Nr3c1* impair HPA-axis communication, leading to depression-like states [28,93,94]. Transcriptomic profiling reveals synaptic dysfunction characterized by the overexpression of oxidative stress and cytokine transcripts (IL-6, TNFα) [95,96,97]. Pharmacological modulation of epigenetic regulators influences stress-responsive and plasticity-related gene networks, including BDNF and HPA-axis-associated pathways, producing changes in depressive-like behaviors [98,99,100]. miRNA-mediated modulation of BDNF signaling is an important mechanism underlying stress-induced depressive behaviors [101,102,103].

Comparative analysis of depression models reveals important conflicts in the literature. While CSDS in C57BL/6J mice reliably produces social avoidance and reduced sucrose preference associated with BDNF downregulation and histone deacetylation, maternal separation models produce heterogeneous outcomes: some studies report resilience-promoting effects of early maternal separation on HPA-axis reactivity, while others report increased vulnerability to subsequent stress [98]. These opposing outcomes are likely to reflect differences in timing, duration, and genetic background. Additionally, some studies report hypomethylation of Nr3c1 in early separation paradigms versus hypermethylation in chronic adult stress paradigms—challenging simple unidirectional models of stress–epigenome interaction and suggesting developmental-stage-specific epigenetic responses.

Regarding model validity, the CSDS model has strong face validity (social withdrawal, anhedonia) and reasonable construct validity. Predictive validity is notably strong—susceptible (but not resilient) mice respond to chronic antidepressant treatment, one of the strongest predictive validity demonstrations in any depression model. Learned helplessness models also show good predictive validity. The main limitations are near-universal use of male rodents and the question of whether rodent social defeat captures the rumination, cognitive distortions, and vegetative symptoms that are central to human MDD.

### 3.6. Animal Models of Rett Syndrome

MECP2 gene mutations directly cause Rett syndrome, illustrating the influence of a single epigenetic regulator on neuronal functioning. Lack of MECP2 protein alters the chromatin structure and lowers histone acetylation, leading to dysregulation of synaptic, mitochondrial, and interneuron-related gene expression [104,105,106]. Many long genes involved in dendritic spine development, synaptic connections, and cognitive processes are differentially expressed in MECP2-null mice, demonstrating pervasive transcriptional dysregulation that primarily impacts neuronal genes with long genomic length and high regulatory complexity [24,107,108]. Partial restoration of MECP2 expression in distinct neuron groups reverses behavioral abnormalities, suggesting that cell-type-specific epigenetic therapies are the most promising [25,109,110].

Within the Rett syndrome model literature, a key molecular debate concerns the relative contribution of different neuron types to the disease phenotype. Studies restoring MeCP2 specifically in cholinergic neurons achieved partial rescue of locomotion and specific phenotypes [110], while restoration in GABAergic neurons rescued a broader range, including breathing deficits and social impairments [30]. This has led to an ongoing debate about whether GABAergic or cholinergic interneuron dysfunction is primary in Rett pathophysiology. Additionally, transcriptomic analyses comparing MeCP2-null brain regions report partially inconsistent gene expression signatures: some studies emphasize the downregulation of long-gene expression programs while others highlight the upregulation of immune and inflammatory transcripts as an equally prominent feature [24,25]. These discrepancies may reflect the brain region analyzed, animal age, and specific MeCP2 allele used.

The MeCP2-null mouse models have the highest construct validity of any model discussed in this review. MeCP2 mutations causally produce Rett syndrome in both mice and humans, and the neurological phenotype, including stereotyped hand movements and breathing irregularities, is faithfully recapitulated. Face validity is therefore excellent. Predictive validity is promising; restoration of MeCP2 expression in adult mice rescues multiple phenotypes, directly motivating gene therapy approaches (scAAV9-MECP2), several of which are in early-phase clinical trials. A key limitation is that most mechanistic studies used male hemizygous MeCP2-null mice. While Rett syndrome predominantly affects females (heterozygous MECP2 mutation carriers), the mosaic pattern of X-inactivation in heterozygous females is not well-modeled by hemizygous males.

### 3.7. Convergent Molecular Pathways Across Disorders

Common inhibitory control deficiencies among various psychiatric diseases are supported by meta-analytic findings from neuropsychiatric research [111]. Seizure phenotypes in animal models are associated with the same molecular processes observed in Rett syndrome, schizophrenia, depression, and ASD. Synaptic plasticity changes, neural inhibition, mitochondrial dysfunction, neuroinflammation, and differences in stress hormone pathways are among the shared mechanisms [33,78,112,113,114]. Immunological signaling pathways such as cytokine-driven modification of immune cell phenotypes may indirectly influence neuroinflammatory mechanisms [115].

The synthesis of findings across the four disorders covered in this review supports two specific conceptual conclusions. First, the convergence of epigenetic dysregulation across ASD, schizophrenia, depression, and Rett syndrome is disproportionately concentrated at inhibitory interneuron-specific loci—particularly PV+ interneuron transcripts, GAD67, and RELN promoters—suggesting a shared circuit-level vulnerability in inhibitory network function that may precede disorder-specific behavioral divergence [116]. While this convergence does not imply a common etiology, it points toward shared circuit-level entry points that could be therapeutically targeted across conditions. Second, the heterogeneity of epigenetic findings across studies is itself biologically informative, rather than a methodological artifact. The epigenome is intrinsically context-dependent—reflecting the developmental stage, cell type, brain region, sex, and prior experience of the organism—arguing against single-biomarker approaches and instead supporting cell-type-resolved, multi-omics, and longitudinal therapeutic targeting strategies.

### 3.8. Advanced Tools and Multi-Omics Approaches

Single-cell RNA sequencing identifies cell-type-specific transcriptional landscapes, revealing distinctions that were previously concealed by bulk tissue research [116,117,118]. Spatial transcriptomics approaches enable the visualization of gene expression and gene enrichment information with anatomical resolution [119,120,121]. Combining ATAC-seq and ChIP-seq creates maps of epigenetic regulatory networks governing gene expression [122,123,124]. Recent computational frameworks, such as super-enhancer regulatory network analysis tools, enable an integrative interpretation of transcriptomic and epigenomic datasets across human and mouse models [125]. Machine-learning techniques increasingly forecast DNA methylation patterns from large datasets [126]. Multimodal datasets combining proteomics, metabolomics, transcriptomics, and epigenomics have facilitated the formation of system networks containing cognitive and psychiatric characteristics, simplifying the identification of treatment targets. Together, these approaches allow for analysis of cell-type- and circuit-specific epigenetic and transcriptomic changes underlying behavioral phenotypes in animal models.

### 3.9. Therapeutic Perspectives

**Figure 6:**
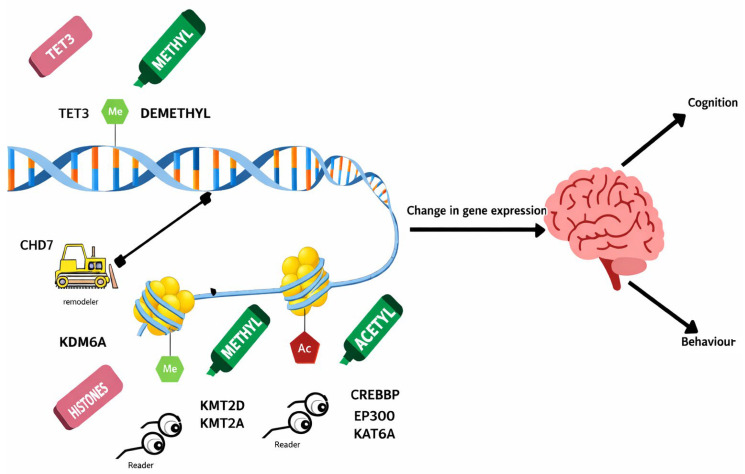
Epigenetic tools and regulators controlling gene expression and brain function. DNMT3A, DNA methyltransferase 3A; TET3, ten-eleven translocation methylcytosine dioxygenase 3; KDM6A, lysine demethylase 6A; KMT2D and KMT2A, lysine methyltransferase 2D and 2A; CREBBP, CREB-binding protein; EP300, E1A-binding protein p300; KAT6A, lysine acetyltransferase 6A; and CHD7, chromodomain helicase DNA-binding protein 7.

Therapeutic opportunities include non-coding RNA-based interventions, pharmacological modulation of epigenetic enzymes such as histone deacetylases and DNA methyltransferases, and CRISPR-based epigenome editing techniques that allow for locus- and cell-type-specific regulation of disease-relevant genes, as shown in Figure 6 [127]. Natural substances like chinonin with epigenetic modulatory qualities are increasingly recognized as potential neuroprotective agents [128]. In preclinical trials, the effectiveness of HDAC inhibitors, DNMT inhibitors, LSD1 modulators, and miRNA mimics in correcting both molecular and behavioral impairments has been extensively established, as shown in Table 2 [129].

**Table 2:** Clinical translation status of major epigenetic and transcriptomic therapeutic strategies for neuropsychiatric disorders, including preclinical evidence, clinical trial status, documented failures, and pathways forward.

Therapeutic Class	Preclinical Evidence	Clinical Translation Status	Key Failures/Challenges	Pathway Forward
HDAC Inhibitors (vorinostat, valproate, romidepsin)	Restore BDNF chromatin accessibility; rescue social/cognitive deficits in depression and ASD rodent models [29,47,49]	Valproate approved for epilepsy/bipolar; evaluated in SCZ Phase II—no superiority over antipsychotics for cognition; vorinostat oncology-approved; neuropsychiatric trials in early phase	Lack of HDAC-isoform specificity causes off-target epigenome-wide effects; limited CNS penetration for non-valproate inhibitors; failure to show cognitive benefit in SCZ trials	Next-generation isoform-selective inhibitors (HDAC2-specific) with improved CNS profiles; PET biomarkers for target engagement
DNMT Inhibitors (5-azacytidine, RG108)	Reverse aberrant promoter hypermethylation (GAD67, RELN) in SCZ and depression models; improve interneuron marker expression	FDA-approved for MDS/AML; CNS neuropsychiatric use investigational; no approved CNS indication	Genome-wide demethylation risks off-target gene activation; toxicity at CNS-efficacious doses; no validated CNS biomarker for dose optimization	Locus-specific demethylation via CRISPR-dCas9-TET fusions for targeted epigenome editing
miRNA-Based Therapies (mimics/antagomirs)	miR-132 and miR-134 modulation improves synaptic plasticity in ASD and depression models; miRNA-BDNF axis corrects stress-induced depressive phenotypes [102]	Early-phase trials in oncology and cardiovascular; no approved CNS neuropsychiatric indication; CNS delivery remains primary obstacle	BBB limits systemic delivery; intrathecal delivery invasive; off-target RNA silencing; immunogenicity of mimics	Exosome-mediated and lipid nanoparticle (LNP)-based CNS delivery systems currently in development
CRISPR Epigenome Editing (dCas9-DNMT, dCas9-TET, dCas9-HDAC)	Locus-specific methylation/acetylation editing at MECP2, RELN, SHANK3 loci restores transcriptional activity in cell and mouse models [130,131]	Preclinical; scAAV9-MECP2 gene therapy trials for Rett syndrome in early clinical phase	Delivery vehicle immunogenicity; off-target editing; MECP2 dosage exquisitely sensitive (overexpression → MECP2 duplication syndrome)	Cell-type-specific AAV capsids with interneuron-selective promoters; base/prime editing to correct specific pathogenic variants
Cell-Type-Specific Epigenetic Rescue	MeCP2 restoration in GABAergic neurons rescues multiple Rett phenotypes; selective HDAC2 modulation in PV+ interneurons corrects E/I imbalance in SCZ models [30]	Indirect clinical precedent via CAR-T concepts; no direct neuropsychiatric application yet	Lack of cell-type-selective CNS delivery in vivo; immunological considerations for viral vectors	Combinatorial promoter + enhancer AAV strategies for cell-type specificity; interneuron-targeting CNS delivery vehicles

Precision targeting of cognitive impairment using pathway-selective receptor agonism represents an emerging precision medicine strategy [132]. Despite the promise of CRISPR-based epigenome editing, important obstacles remain at the translational level: delivery across the blood–brain barrier with sufficient efficiency and safety, off-target editing effects at near-cognate genomic sites, the limited cell-type specificity of current delivery systems, and long-term safety concerns [130,131,133,134]. Additionally, broader cell-based strategies have highlighted the complexity of achieving circuit-specific effects [105,135]. Digital and virtual reality interventions are becoming more popular as supplemental methods to improve cognitive function in neuropsychiatric disorders [136]. It is anticipated that precision techniques relying on cell-specific epigenetic regulation and multilayer omics-guided drug discovery will eventually result in the creation of individualized therapeutics for neuropsychiatric and cognitive disorders.

## 4. Discussion

Neuroscience and psychology primarily rely on evidence-based advancements in epigenetic and transcriptomic research to understand the molecular genetics of cognitive and psychiatric disorders [69]. The studies synthesized in this review have reached a consensus that long-lasting changes in neural circuits are caused by changes in DNA methylation, histone modification, and the activity of non-coding RNAs [96]. In animal models—particularly those involving rodents exposed to early-life stress, chronic stress, environmental enrichment, or genetic modifications—epigenetic changes brought about by experiences are dependent on cognitive abilities, emotional regulation, and the likelihood of developing psychiatric disorders [15]. For example, stress-induced methylation alterations of genes like BDNF and NR3C1 impair neuronal plasticity and stress hormone control, resulting in behavior resembling human illnesses such as PTSD and depression [102].

Mounting evidence from studies on schizophrenia shows that important inhibitory indicators including reelin and GAD67 are produced less frequently, while their promoters are more methylated [21]. The epigenetic dysregulation paradigm of decreased inhibitory interneuron maintenance and disrupted E/I balance is supported by all of these data [126]. Specific gene expression changes such as microglial activation, mitochondrial dysfunction, synaptic remodeling, and abnormal pathways of neurodevelopment have been highlighted by transcriptome profiling techniques including RNA sequencing and single-cell approaches [93]. The transcriptome characteristics above highlight the need to consider neuropsychiatric diseases as results of disrupted network-level communication among different brain areas and cell types, rather than as the results of single gene abnormalities [122].

The interaction between nature and nurture is highlighted by comparing these many studies. Early experiences can leave epigenetic “marks” that later affect the cognitive or affective outcomes [7]. Critically, the reversibility of some epigenetic alterations has been demonstrated. Treatment with HDAC inhibitors, environmental enrichment, exercise, and medications have improved behavioral deficiencies and restored altered gene expression in models of human disorders [100].

Even though the field has made great strides, positive findings have been accompanied by significant variation among studies, primarily due to different experimental models, tissues, and epigenomic technology methodologies [99,126]. Still, decreased synaptic plasticity, neuroinflammation, and altered stress signaling are common ways in which the variables interact [115]. The evidence gathered thus far supports the theory that the transcriptome and epigenome are among the factors determining psychological and psychiatric traits and could be used for intervention aimed at changing gene regulation [7,130]. Translational promise for cognitive stratification is provided by emerging neuroimaging biomarkers, such as cortical activity profiles based on fNIRS [132]. AI-powered digital biomarkers may enhance molecular omics in improving treatment monitoring and diagnosis [137].

A critical challenge for the field is the persistence of the translational gap between animal model epigenomics and human disease. Several factors compound this challenge. First, the human brain shows more extensive non-CG methylation (mCH) in post-mitotic neurons than the mouse brain, and the regulatory consequences of this difference for psychiatric disease-relevant gene networks are not fully characterized. Second, the developmental timeline of human brain maturation—particularly prefrontal cortical maturation extending into the third decade of life—means that mouse models of adolescent or adult epigenetic change may not capture developmentally equivalent time points. Third, the vast majority of included studies used male-only or male-predominant cohorts; given the known sex differential in epigenome regulation and in clinical presentations of ASD, schizophrenia, and depression, this represents a systematic blind spot that future studies must address. Fourth, most animal model transcriptomics have been performed on bulk tissue, obscuring the cell-type-specific epigenetic changes that are the most mechanistically relevant. The rapid adoption of single-cell and spatial transcriptomics approaches in 2023–2025 is the most promising technical development for closing this gap.

### 4.1. Advantages

This scoping review integrates studies on epigenetics and transcriptomics across multiple disorders to provide a comprehensive picture of how these mechanisms may influence cognitive and psychiatric outcomes [62]. Using cutting-edge methods including whole-genome methylation profiling, single-cell sequencing, and chromatin accessibility assays, it highlights compelling mechanistic evidence connecting animal to human work [99]. Schizophrenia, depression, autism, and Rett syndrome were among the disorders evaluated, broadly demonstrating the applicability of these pathways [1,57,109]. This review aids in identifying convergent biological pathways that may serve as therapeutic targets by combining molecular and behavioral data from neurocircuits [95,138].

### 4.2. Limitations

Direct comparisons are challenging, because the included studies differ in animal models, treatment stress paradigms, sequencing technology, and behavioral assays [91]. Confounders in human postmortem investigations include age, drug history, and lifestyle factors [97]. The majority of results remain correlational, rather than causal [135]. Women are typically understudied, with most research conducted on male rodents [84]. As a scoping review, this work does not perform a quantitative meta-analysis or pooled synthesis of effect sizes; rather, it maps and characterizes the evidence landscape, which limits the strength of directional conclusions that can be drawn.

### 4.3. Future Prospects

Future omics studies should combine transcriptomics, proteomics, metabolomics, and epigenomics to properly examine disease pathways. Single-cell and spatial transcriptomics will make it possible to identify particular cell types where molecular changes in neural circuits are occurring. Human iPSC-derived neurons and brain organoids, while not replacing animal models, offer a complementary system that captures human genetic and epigenetic variation and bridges the translational gap. Using HDAC inhibitors, DNMT modulators, miRNA-based treatments, and CRISPR-mediated epigenome editing could present a great chance to build tailored epigenetic therapies. The use of peripheral biomarkers in conjunction with sex as a biological variable might facilitate individualized treatment strategies for neuropsychiatric disorders.

## 5. Conclusions

Animal models are invaluable for understanding how epigenetic and transcriptomic pathways alter cognitive and psychiatric traits. Several shared dysregulation-related biological pathways—neuroinflammation, mitochondrial malfunction, synaptic abnormalities, and neuronal inhibition—represent the converging lines of evidence contributing to shared vulnerabilities. A precision medicine approach to restoring neural resilience and improving outcomes in human neurodevelopmental and psychiatric disorders may be made possible by new technologies such as single-cell transcriptomics, spatial mapping, and CRISPR-based techniques. Future work must prioritize longitudinal, multi-region, cell-type-resolved, and sex-inclusive designs—in both animal models and human studies—to fully realize the translational potential of epigenomic and transcriptomic discoveries in neuropsychiatric medicine.

## Figures and Tables

**Table 1:** (**A**). Primary experimental studies (n = 28) illustrating key epigenetic and transcriptomic mechanisms across ASD, schizophrenia (SCZ), depression, and Rett syndrome. All entries specify species, strain, sex, experimental approach, molecular findings, validity profile, and study limitations. (**B**). Key review and conceptual papers (n = 12) providing theoretical frameworks that are foundational to this field, with justification for inclusion in scoping review corpus.

(A)							
**Reference**	**Disorder**	**Animal Model (Species/Strain/Sex)**		**Epigenomic/Transcriptomic Approach**	**Key Molecular Findings**	**Construct/Face/Predictive Validity**	Key Limitations
Peça et al., 2011 [19]	ASD	Shank3 KO mouse; C57BL/6J; male		Behavioral phenotyping; synaptic electrophysiology; protein expression	Loss of Shank3 → striatal dysfunction, impaired social interaction, repetitive behaviors; reduced dendritic spine density; altered glutamatergic signaling	Construct validity high (SHANK3 mutations → Phelan–McDermid syndrome in humans); face validity good; predictive validity partial (mGluR5 rescue in mice failed in clinical trials)	Single genetic locus; does not model polygenic idiopathic ASD; C57BL/6J background may mask phenotypes
Schneider et al., 2007 [20]	ASD	VPA-exposed Wistar rat; prenatal E12.5; male offspring		Behavioral assays; enkephalinergic system analysis; immunohistochemistry	Prenatal VPA disrupts enkephalinergic signaling, alters hedonic tone; produces autism-like behavioral profile including social withdrawal and repetitive behaviors	Face validity moderate; construct validity weak (VPA = one of many heterogeneous ASD causes)	Male only; VPA has broad epigenome-wide effects, making causal pathway isolation difficult
Guidotti et al., 2000 [21]	SCZ	Human postmortem prefrontal cortex (translational benchmark)		Quantitative RT-PCR; in situ hybridization	Decreased RELN and GAD67 in PFC and hippocampus; associated with promoter hypermethylation in SCZ and bipolar disorder	Strong face validity for inhibitory interneuron hypothesis; used to benchmark animal model epigenetic changes	Postmortem only; confounders include antipsychotic history, substance use, PMI
Maze et al., 2010 [22]	Depression	C57BL/6J mice; CSDS protocol; male		ChIP-seq; H3K9me2 profiling; G9a conditional KO	G9a (H3K9 methyltransferase) in nucleus accumbens regulates stress-induced plasticity; H3K9me2 reduction permits maladaptive gene expression	Construct validity moderate; face validity good (social avoidance, anhedonia)	Drug-reward study adapted to stress; male only; predictive validity for antidepressants not directly tested
McGowan et al., 2009 [23]	Depression/early-life stress	Human hippocampal tissue (suicide victims ± childhood abuse)		Bisulfite sequencing; pyrosequencing of NR3C1 promoter	Childhood abuse → increased NR3C1 promoter methylation → reduced GR expression → impaired HPA-axis regulation	First strong human evidence linking early trauma to epigenetic stress circuitry dysregulation	Postmortem only; retrospective abuse history; no cell-type specificity
Lister & Mukamel et al., 2015 [8]	Cross-disorder (brain development)	Mouse frontal cortex + human reference; multiple developmental stages		WGBS; single-cell methylation profiling	Dynamic non-CG methylation (mCH) in post-mitotic neurons contributes to cell-type identity and brain plasticity; neuron-specific methylation landscapes identified	Foundational for understanding brain-region and cell-type specificity of epigenetic marks	Predominantly correlational; human mCH patterns differ quantitatively from mice
Bajikar et al., 2025 [24]	Rett syndrome	MeCP2-null mice; C57BL/6J; male and female; adult inducible		scRNA-seq; ATAC-seq; chromatin accessibility profiling	Acute MeCP2 loss triggers transcriptional and chromatin changes preceding overt symptoms; long neuronal genes are preferentially dysregulated	High construct validity (MECP2 mutations cause Rett syndrome); face validity strong	Acute model may not fully recapitulate developmental progression; predominantly male in early analyses
Moore et al., 2025 [25]	Rett syndrome	MeCP2-null and hypomorphic mice; C57BL/6J		scRNA-seq; bisulfite sequencing; long-gene expression profiling	MeCP2 and non-CG DNA methylation cooperate to stabilize expression of long genes, distinguishing closely related neuron types	High construct validity; demonstrates cell-type-specific mechanism beyond global repression	Male-biased cohort; genetic homogeneity may not capture human allelic diversity
Won et al., 2012 [26]	ASD	Shank2 KO mouse; C57BL/6N; male		Behavioral battery; electrophysiology; NMDA receptor pharmacology	Autistic-like social behavior improved by restoring NMDA receptor function; distinct glutamatergic mechanism from Shank3	Good face validity; demonstrates molecular heterogeneity within ASD; predictive validity supported by partial pharmacological rescue	Single gene; male only; three-chamber test may not fully capture human social cognition
Moore et al., 2006 [27]	SCZ	MAM-treated Sprague-Dawley rat; E17 injection; male		Behavioral neuroscience; electrophysiology; neurodevelopmental neuropathology	MAM disrupts cortical lamination, causes hypofrontality, reduces prefrontal synaptic gene expression—mimics SCZ neurodevelopmental features	Moderate-to-high construct validity (neurodevelopmental disruption); good face validity (PPI deficits, cognitive impairment)	Male only; laminar disruption differs from human SCZ anatomical patterns
Rapanelli et al., 2022 [28]	ASD/SCZ	Shank3 and DISC1 mutant mice; C57BL/6J; both sexes		LSD1 ChIP; behavioral rescue; pharmacology	Targeting histone demethylase LSD1 ameliorates behavioral abnormalities and partially restores gene expression in both ASD and SCZ models	High translational potential; identifies LSD1 as shared therapeutic target; both sexes included	Long-term safety of LSD1 inhibition not fully characterized
Covington et al., 2009 [29]	Depression	C57BL/6J mice; CSDS; male		HDAC inhibition; ChIP; behavioral assays	HDAC inhibitors restore chromatin accessibility at BDNF promoter; improve depressive-like behavior; antidepressant-like profile	Good construct and face validity; predictive validity partially supported (HDAC inhibitors show benefit in treatment-resistant depression trials)	Male only; HDAC inhibition is not isoform-specific; off-target epigenome effects
Ure et al., 2016 [30]	Rett syndrome	MeCP2 conditional rescue mice; GABAergic-specific; C57BL/6J		Cre-lox conditional rescue; behavioral battery; EEG	Restoration of MeCP2 in GABAergic neurons rescues multiple Rett-like phenotypes including breathing, social, and motor deficits	High construct validity; demonstrates cell-type-specific sufficiency of MeCP2 in inhibitory neurons	Developmental timing of restoration not modeled; sex: mixed
**(B)**							
**Reference**	**Scope**		**Key Conceptual Contribution**		**Limitations of This Source**	**Justification for Inclusion**	
Jaenisch & Bird, 2003 [6]	Cross-disorder		Epigenetic control integrates environmental signals into the genome; foundational mechanisms defined		No primary data; mechanisms described predate single-cell resolution—limits direct application to circuit-level psychiatric analyses	Landmark paper; defines core epigenetic concepts that are foundational to the entire field	
Reik et al., 2001 [31]	Developmental epigenetics		Epigenetic reprogramming required for early mammalian development; key vulnerability windows identified		Developmental stage-specific; does not address post-mitotic neurons or psychiatric disease models	Introduced concept of reprogramming windows that are critical to prenatal exposure models	
Zoghbi & Bear, 2012 [3]	ASD		Synaptic dysfunction (mGluR5, SHANK, FMRP) underlies autism and intellectual disability		Focused on syndromic autism; broader idiopathic ASD heterogeneity not covered	Established synaptic plasticity as core diagnostic and therapeutic target in ASD	
Day & Sweatt, 2011 [4]	Memory/cognition		Epigenetic mechanisms (DNA methylation, histone modification) regulate memory formation and cognition		Primarily animal-based evidence; human applicability not fully established at time of publication	Foundational framework for epigenetic basis of learning and memory	
LaSalle, 2013 [32]	ASD		Epigenomic disturbances link genetic predisposition and environmental exposure in autism		Limited clinical translation; mechanistic pathways not fully resolved at time of writing	Synthesized emerging epigenetic risk pathways in ASD	
Liu C et al., 2018 [7]	Depression, SCZ, BD		Methylation alterations associated with depression, bipolar disorder, and schizophrenia		Heavy reliance on associative data; causal inference limited	Connects methylation patterns with psychiatric symptom profiles across major disorders	
Thomas R et al., 2010 [2]	SCZ		Brain circuit disruption in SCZ framed as a neurodevelopmental illness with epigenetic contributions		Broad conceptual review; some mechanistic claims since refined	Provides integrative model of SCZ pathology guiding subsequent animal model design	
Dominguez-Oliva et al., 2023 [15]	Cross-disorder		Comprehensive contemporary evaluation of animal model utility for neurodevelopmental and psychiatric pathways		Species and strain differences (rat vs. mouse; inbred vs. outbred) limit direct comparisons	Detailed evaluation of model benefits and limitations for neuropsychiatric research	
Michael C.J. et al., 2024 [14]	Cross-disorder		Perspective: animal models remain irreplaceable for in vivo circuit-level neuroscience; discusses 3Rs (Replacement, Reduction, Refinement) framework		No primary data; ‘ethical considerations’ in original table referred to general 3Rs principles, NOT specific violations—misleadingly worded in prior version	Contemporary justification for translational animal studies; directly contextualizes all primary studies in this review	
Piazzi et al., 2023 [12]	Cross-disorder		NGS currently has a limited ability to identify RNA edits and alternative splicing at transcriptome scale		Technology-specific review; some limitations are being actively overcome by long-read sequencing	Identifies important gaps and future directions for transcriptomic tools in psychiatric research	
Cattane et al., 2022 [33]	Cross-disorder		Animal models of mental illness: molecular brain and peripheral biomarkers associated with early-life stress and immune challenges		Heterogeneity of stress paradigms and species limits cross-study comparisons; male-biased	Strong synthesis of translational mechanisms linking preclinical models to psychiatric biomarkers	
Irfan A et al., 2018 [5]	Multiple neurological		Numerous neurological disorders linked to epigenetic dysregulation; clinical translation perspective		Broad review with limited disease-specific depth; mechanisms not fully distinguished across conditions	Clinical translation perspective connecting epigenetic dysregulation across neurological conditions	

## Data Availability

No new data were created or analyzed in this study. Data sharing is not applicable to this article.

## Supplementary Materials

The following supporting information can be downloaded at: https://www.mdpi.com/article/10.3390/cimb48040425/s1.

## Abbreviations

ASD	Autism Spectrum Disorder
SCZ	Schizophrenia
PTSD	Post-Traumatic Stress Disorder
HPA axis	Hypothalamic–Pituitary–Adrenal axis
MIA	Maternal Immune Activation
Poly I:C	Polyinosinic:polycytidylic acid
MAM	Methylazoxymethanol acetate
VPA	Valproic Acid
DNMT	DNA Methyltransferase
HDAC	Histone Deacetylase
LSD1	Lysine-Specific Demethylase 1
CRISPR	Clustered Regularly Interspaced Short Palindromic Repeats
RNA-seq	RNA Sequencing
scRNA-seq	Single-Cell RNA Sequencing
ATAC-seq	Assay for Transposase-Accessible Chromatin Sequencing
ChIP-seq	Chromatin Immunoprecipitation Sequencing
iPSC	Induced Pluripotent Stem Cell
lncRNA	Long Non-Coding RNA
miRNA	MicroRNA
circRNA	Circular RNA
A-to-I	Adenosine-to-Inosine (RNA editing)
PRISMA-ScR	Preferred Reporting Items for Systematic Reviews and Meta-Analyses extension for Scoping Reviews
CAMARADES	Collaborative Approach to Meta-Analysis and Review of Animal Data from Experimental Studies
E/I	Excitatory–Inhibitory (balance)
PV+	Parvalbumin-positive (interneurons)
3Rs	Replacement, Reduction, Refinement
CSDS	Chronic Social Defeat Stress
WGBS	Whole-Genome Bisulfite Sequencing
