# Supplementary material for: Epigenetic and Transcriptomic Pathways Underlying Animal Models of Cognitive and Psychiatric Disorders: A Scoping Review
Source: Curr Issues Mol Biol. 2026 Apr 21;48(4):425. doi: 10.3390/cimb48040425 (PMC13115134; doi:10.3390/cimb48040425)
Supplement: Supplementary file 1 [file cimb-48-00425-s001.zip › cimb-4215910-supplementary.pdf]

Table S1-Summary. CAMARADES quality appraisal of 63 primary experimental studies: aggregate results across six criteria. (†n (%) = number and percentage of studies meeting each criterion fully.)

CAMARADES Criterion	Met: n (%)†	Partially met: n (%)	Not met / not reported: n (%)	Notes
1. Use of randomization in group allocation	41 (65%)	8 (13%)	14 (22%)	Most genetic model studies report random allocation; environmental model studies (VPA, CSDS) less consistently report this
2. Blinded outcome assessment	28 (44%)	12 (19%)	23 (37%)	Blinding most consistently reported in behavioral phenotyping studies; molecular studies (ChIP-seq, RNA-seq) rarely report blinding at analysis stage
3. Sample size justification / power calculation	18 (29%)	5 (8%)	40 (63%)	Under-reporting is a field-wide issue; most studies state group sizes without formal power calculation
4. Conflict of interest disclosure	58 (92%)	0 (0%)	5 (8%)	High compliance; most journals

CAMARADES Criterion	Met: n (%)†	Partially met: n (%)	Not met / not reported: n (%)	Notes
				require COI statement
5. Animal housing and welfare conditions	47 (75%)	9 (14%)	7 (11%)	Generally well-reported for genetic models; older studies (pre-2010) less detailed
6. Appropriate statistical methods with effect size reporting	39 (62%)	14 (22%)	10 (16%)	Use of appropriate tests generally adequate; effect size reporting (Cohen's d, η^2) inconsistent, particularly in studies pre-2015

Supplementary Table S2: Detailed documentation of study screening, eligibility assessment, and evidence categorization is provided in to improve transparency and reproducibility of the study selection process.

Study (First Author, Year)	Article Type	Disorder/Topic	Model System / Population	Screening Outcome	Reason for Exclusion (if excluded)	Evidence Category
Peça et al., 2011	Primary research	Autism spectrum disorder	Shank3 knockout mouse	Included	–	Primary experimental
Schneider et al., 2007	Primary research	Autism spectrum disorder	Prenatal VPA-exposed rat	Included	–	Primary experimental
Guidotti et al., 2000	Human molecular study	Schizophrenia	Human postmortem PFC	Included	–	Translational
Maze et al., 2010	Primary research	Depression	Chronic social defeat stress mouse	Included	–	Primary experimental
McGowan et al., 2009	Human epigenetic study	Depression / early-life stress	Human hippocampus	Included	–	Translational
Won et al., 2012	Primary research	Autism spectrum disorder	Shank2 knockout mouse	Included	–	Primary experimental
Rapanelli et al., 2022	Primary research	ASD / schizophrenia	Genetic mouse models	Included	–	Primary experimental
Ure et al., 2016	Primary research	Rett syndrome	MeCP2 conditional mouse	Included	–	Primary experimental
Example Review Study 1	Narrative review	Neuropsychiatric epigenetics	Not applicable	Excluded	Secondary literature used only for background context	Secondary
Example Review Study 2	Systematic review	Epigenetics in ASD	Not applicable	Excluded	Did not provide primary experimental data	Secondary
